# Determinants of antenatal care and skilled birth attendance services utilization among childbearing women in Guinea: evidence from the 2018 Guinea Demographic and Health Survey data

**DOI:** 10.1186/s12884-020-03489-4

**Published:** 2021-01-03

**Authors:** Bright Opoku Ahinkorah, Abdul-Aziz Seidu, Ebenezer Agbaglo, Collins Adu, Eugene Budu, John Elvis Hagan, Thomas Schack, Sanni Yaya

**Affiliations:** 1 School of Public Health, Faculty of Health, University of Technology Sydney, Ultimo, Australia; 2 Department of Population and Health, University of Cape Coast, Cape Coast, Ghana; 3 Department of English, University of Cape Coast, Cape Coast, Ghana; 4 Department of Health Promotion and Disability Studies, Kwame Nkrumah University of Science and Technology, Kumasi, Ghana; 5 Department of Health, Physical Education, and Recreation, University of Cape Coast, Cape Coast, Ghana; 6 Neurocognition and Action-Biomechanics-Research Group, Faculty of Psychology and Sport Sciences, Bielefeld University, Bielefeld, Germany; 7 School of International Development and Global Studies, University of Ottawa, Ottawa, Canada; 8 The George Institute for Global Health, The University of Oxford, Oxford, United Kingdom

**Keywords:** Antenatal care, Skilled birth attendance, Guinea, Public Health, Global Health

## Abstract

**Background:**

Globally, maternal health remains a major priority. Most of maternal deaths globally occur in sub-Saharan Africa, with most of these deaths linked to lack of access to antenatal care and skilled assistance during delivery. This study assessed the determinants of antenatal care and skilled birth attendance services utilization among childbearing women in Guinea.

**Methods:**

Data for this study were obtained from the 2018 Guinea Demographic and Health Survey (GDHS). Data of 4,917 childbearing women were considered as our analytical sample. The outcome variables for the study were utilization of antenatal care and skilled birth attendance. Analysis was carried out using chi-square tests and multivariable logistic regression.

**Results:**

The results showed that women aged 15-24 (AOR=1.29, CI=1.03-1.62), women who had secondary/higher level of education (AOR=1.70, CI=1.33-2.19), and those whose partners had secondary/higher level of education (AOR=1.46, CI=1.22-1.75), women in the richest wealth quintile (AOR=5.09, CI=3.70-7.00), those with planned pregnancies (AOR=1.50, CI=1.23-1.81), Muslim women (AOR=1.65, CI=1.38-2.12), those who take healthcare decisions alone (AOR=1.53, CI=1.24-1.89), and those who listened to radio less than once a week (AOR= 1.30, CI=1.10-1.53) had higher odds of antenatal care uptake. Also, women with secondary/higher level of education (AOR=1.83, CI=1.25-2.68), those whose partners had secondary/higher level of education (AOR=1.40, CI=1.11-1.76), those in the richest wealth quintile (AOR=10.79, CI=6.64-17.51), those with planned pregnancies (AOR=1.25, CI=1.03-1.52), Christian women (AOR=4.13, CI=3.17-5.39), those living in urban areas (AOR=3.00, CI=2.29-3.94), women with one birth (AOR= 1.58, CI=1.20-2.06), those who take healthcare decisions alone (AOR=1.87, CI=1.46-2.39), those who read newspaper at least once a week (AOR= 1.19, CI=1.01-1.40), those who watched television at least once week (AOR=1.69, CI=1.30-2.19), and those in female-headed households (AOR=1.52, CI=1.20-1.92) were more likely to utilize the services of skilled birth attendants.

**Conclusion:**

The study proved that various socio-economic and contextual factors influence antenatal care and skilled birth attendance in Guinea. These findings suggest the need to design community-based interventions (e.g., miniature local ANC clinics, early screening services) that prioritize women’s education and vocational training, media accessibility, especially among the poor, and those residing in rural settings. Such interventions should not ignore the influence of other socio-cultural norms that hinder the utilization of antenatal care and skilled birth attendance services in Guinea.

## Background

Globally, maternal healthcare remains a very important issue in public health. For pregnancy, childbirth, and post-delivery periods, healthcare services are absolutely necessary for the well-being and survival of mothers and children [1]. Antenatal care (ANC) and delivery care, especially the use of skilled birth attendants during delivery, are of extreme importance in cutting down maternal deaths which claim the lives of many women across the world [1]. ANC is a widely recognized strategy for reducing maternal morbidity and mortality. It also represents an entry point for the integrated use of both facility-based delivery and skilled birth attendants [2]. Receiving antenatal care at least four times, as recommended by World Health Organization (WHO), increases the likelihood of receiving effective maternal health interventions during antenatal visits [3].

Most of maternal deaths globally occur in low- and middle-income countries (LMICs), with majority of the deaths recorded in sub-Saharan Africa [4], due to lack of access to quality ANC and skilled birth attendance (SBA) during delivery [5]. Guinea is one of the countries in sub-Saharan Africa with little advancement in reducing maternal mortality. Over the years, there have been attempts to institute programs aimed at reducing maternal mortality in Guinea. Such programs have focused on routine identification, notification, quantification, and determination of causes and avoidability of maternal death, as well as the use of this information to respond through actions that will prevent future deaths [6]. Despite these measures, Guinea’s maternal mortality ratio was estimated at 576 per 100,000 live births in 2017 [7]. This ratio remains too high if the country is to attain target one of the Sustainable Development Goal Three (SDG 3), which aims at reducing the global maternal mortality ratio to as low as 70 per 100,000 live births by 2030.

Previous studies in sub-Saharan Africa as a whole, e.g., [8, 9] and in specific countries such as Malawi [5], Ethiopia [10], Ghana [11], and Nigeria [12, 13] made efforts to build on the understanding of factors influencing maternal healthcare utilization. These studies identified education, mother’s age, place of residence, household income and employment status as determinants of maternal healthcare utilization, yet, for every country, fluctuations in maternal mortality were recorded. This trend clearly shows that findings from previous studies done in other countries in sub-Saharan Africa are inconclusive and may require further enquiry into some context -specific issues around ANC and SBA in a country like Guinea, noted for its high maternal mortality ratio. There seems to be a paucity of empirical research linking multiple predictors of ANC and SBA in Guinea. Understanding the association between these interrelated health constructs can support the design of appropriate or strategic interventions and policy modifications for key sections’ of Guinea’s population to help promote healthy maternal outcomes for women and children. Additionally, extending literature on the utilization of ANC and SBA services in Guinea that previous studies have ignored would be quite essential. This present study therefore assessed maternal healthcare utilization and its associated factors among childbearing women in Guinea, with particular focus on ANC and SBA services.

### Methods

## Data Source

Data for this study were obtained from the 2018 Guinea Demographic and Health Survey (GDHS). Specifically, the women’s file was used. GDHS is part of a number of surveys obtained from the MEASURE DHS Program, which provides information on a number of population and health issues such as maternal healthcare services utilization. Data of 4,917 childbearing women who had complete information on the variables of interest were considered as the analytical sample. The GDHS utilized a multi-stage, stratified sampling procedure for selecting all eligible women for interviews from households, which were considered as sampling units. Detailed information concerning the methodology, instruments, pretesting of the instruments, training, and recruitment of enumerators can be found in the final report [14] and avaliable online at the following weblink: https://dhsprogram.com/publications/publication-FR353-DHS-Final-Reports.cfm.

## Study variables

### Outcome variables

Two outcome variables were considered in this study: utilization of ANC and SBA. With ANC, women who had live births were asked about the number of antenatal visits they made during their recent pregnancy. The number of ANC visits was coded as < 4 visits = 0 and ≥ 4 visits = 1. The reason for this coding was in line with the WHO’s recommendation of at least four ANC visits [3]. Previous studies on ANC have also adopted this categorization [15–19]. Women who had delivered were also asked whether their delivery was assisted by a skilled health personnel (i.e., doctor, nurse, midwife, or trained village health worker). Those who indicated ‘Yes’ were considered as having ‘skilled birth attendance’ and this was coded as ‘1’ while women who were attended by traditional birth attendants/parents/friends/neighbours were considered as not having skilled birth attendance and were given the code ‘0’.

## Independent variables

The independent variables that were included in the estimations to assess the predictors of ANC and SBA services were age, mother’s level of education, partner’s level of education, wealth quintile, pregnancy intention, religion, marital status, place of residence, parity, employment status, healthcare decision-making, frequency of reading newspaper/magazine, frequency of listening to radio, frequency of watching television, and sex of household head (see Table 1). These variables were chosen due to their significant associations with ANC and SBA in previous studies in sub-Saharan Africa [15–19]. Healthcare decision-making was derived from the DHS question that asked women who decides on their healthcare. The responses were respondent alone, respondent and husband/partner, husband/partner alone, someone else and other. These were recoded into alone = 1, not alone = 2.

**Table 1:** Distribution of antenatal care and skilled birth attendance by socio-demographic characteristics of childbearing women in Guinea (Weighted *N* = 4,917)

Variables	Frequency (N)	Percentage (%)	ANC (35.3%)	*p*-value	SBA (58.9%)	*p*-value
**Age**				*p* < 0.001		*p* < 0.01
15–24	1,324	26.9	38.8		60.1	
25–34	2,254	45.8	34.6		57.6	
35+	1,339	27.2	31.3		53.4	
**Mother’s level of education**				*p* < 0.001		*p* < 0.001
No education	3,852	78.4	30.1		50.8	
Primary	513	10.4	42.1		69.8	
Secondary/Higher	552	11.2	61.4		89.9	
**Partner’s level of education**				*p* < 0.001		*p* < 0.001
No education	3595	73.1	29.6		50.1	
Primary	349	7.1	40.1		63.3	
Secondary/Higher	973	19.8	52.8		81.7	
**Wealth quintile**				*p* < 0.001		*p* < 0.001
Poorest	1,205	24.5	18.6		30.9	
Poorer	1,116	22.7	26.3		42.7	
Middle	978	19.9	31.6		55.4	
Richer	920	18.7	48.9		82.3	
Richest	699	14.2	63.5		95.9	
**Pregnancy intention**				*p* < 0.001		*p* < 0.01
Unintended	702	14.3	28.8		51.7	
Planned	4215	85.7	35.9		58.0	
**Religion**				*p* < 0.001		*p* < 0.001
Christianity	515	10.5	25.3		78.3	
Islam	4320	87.9	35.8		54.9	
Others	82	1.7	26.5		77.6	
**Marital status**				*p* < 0.05		*p* < 0.001
Married	4817	98.0	34.6		56.7	
Cohabiting	100	2.0	46.3		80.5	
**Place of residence**				*p* < 0.001		*p* < 0.001
Urban	1341	27.3	53.5		91.8	
Rural	3576	72.7	27.8		44.0	
**Parity**				*p* < 0.001		*p* < 0.001
One birth	771	15.7	40.6		69.0	
Two births	880	17.9	37.4		59.8	
Three births	925	18.8	34.8		58.1	
Four or more births	2,341	47.6	32.0		51.9	
**Employment status**				*p* > 0.05		*p* < 0.05
Not working	1,208	24.6	35.2		59.6	
W orking	3,709	75.4	34.7		56.2	
**Health care decision making**				*p* < 0.01		*p* < 0.001
Alone	498	10.1	42.2		67.0	
Not alone	4,419	89.9	34.1		56.1	
**Frequency of reading newspaper/magazine**				*p* < 0.001		*p* < 0.001
Not at all	4,667	94.9	33.6		55.3	
Less than once a week	145	3.0	58.6		90.6	
At least once a week	105	2.1	62.5		95.2	
**Frequency of listening to radio**				*p* < 0.001		*p* < 0.001
Not at all	2,038	41.5	28.9		49.6	
Less than once a week	1,372	27.9	40.9		62.9	
At least once a week	1,507	30.6	37.5		62.2	
**Frequency of watching television**				*p* < 0.001		*p* < 0.001
Not at all	3,066	62.4	27.2		44.3	
Less than once a week	876	17.8	41.0		71.6	
At least once a week	975	19.8	54.7		86.7	
**Sex of household head**				*p* < 0.01		*p* < 0.001
Male	4,419	88.9	34.2		55.9	
Female	498	110.1	40.2		68.2	

**Source: 2018 Guinea Demographic and Health Survey**

### Data analysis

The first step of the analyses involved the use of frequency tabulations to describe the proportions of all the explanatory variables. The second step was a bivariate chi-square analysis on the distribution of ANC and SBA across the independent variables. This analysis was followed by a multivariable logistic regression analysis to examine the predictors of ANC and SBA. All the variables that showed statistical significance with ANC and SBA at p < 0.05 were considered for the multivariable logistic regression analysis. The results were presented using adjusted odds ratio (AOR) with their corresponding 95% confidence intervals (CIs). Before conducting the multivariable logistic regression analysis, a multi-collinearity test was carried out among all the statistically significant variables to determine whether there was evidence of high collinearity among them. Using the variance inflation factor (VIF), the multicollinearity test showed that there was no evidence of collinearity among the explanatory variables (Mean VIF = 1.41, Maximum VIF = 2.33, Minimum = 1.02**)**. Goodness-of-fit of the logistic regression models were assessed using Pseudo R^2^. Data cleaning, management, and analysis were carried out using Stata version 14.2 (StataCorp, College Station, Texas, USA). Applied sample weight (v005/1,000,000) to correct for over-and under-sampling was employed and the SVY command was used to cater for the complex survey design and generalizability of the findings.

## Results

### Distribution of ANC and SBA by socio-demographic characteristics of childbearing women in Guinea

Table 1 presents the results on the distribution of ANC and SBA by socio-demographic characteristics of childbearing women in Guinea. The results indicate that 45.9% of study participants were aged 25–34 and 78.4% had no formal education. A quarter (24.5%) of the women were in the poorest wealth quintile, as compared to 14.2% who were in the richest wealth quintile. The majority of the women (85.7%) had planned pregnancies, 87.9% of the women were Muslims, 75.5% were working, and almost all (98%) of them were married. Additionally, 72.7% were in the rural areas, 47.6% had parity 4 or more and 88.9% were in male-headed households. With media exposure, 94.9% of the sample did not read newspaper or magazine at all, 41.5% and 62.4% respectively did not listen to radio or watched television at all. The chi-square analysis also showed that all the independent variables apart from employment status showed statistically significant associations (p < 0.001) with ANC services utilizations while all of them showed statistically significant associations (*p* < 0.001) with SBA services utilization.

### Predictors of ANC and SBA utilization among childbearing women in Guinea

Table 2 shows the results on the predictors of maternal healthcare utilization among childbearing women in Guinea. For ANC utilisation, women aged 15–25 had higher odds (AOR = 1.29, CI = 1.03–1.62) of ANC uptake, compared to those aged 35 + was found. Similarly, women who had secondary/higher level of education (AOR = 1.70, CI = 1.33–2.19) and those whose partners had secondary/higher level of education (AOR = 1.46, CI = 1.22–1.75) had higher odds of ANC uptake were realized. The results further showed that those in the richest wealth quintile (AOR = 5.09, CI = 3.70-7.00), those with planned pregnancies (AOR = 1.50, CI = 1.23–1.81), Muslim women (AOR = 1.65, CI = 1.38–2.12), those who take healthcare decisions alone (AOR = 1.53, CI = 1.24–1.89), and those who listened to radio less than once a week (AOR = 1.30, CI = 1.10–1.53) had higher odds of ANC uptake, compared to those in the poorest wealth quintile, those with unintended pregnancy, Christians, those who do not take healthcare decisions alone, and those who do not listen to radio at all.

**Table 2:** Predictors of antenatal care and skilled birth attendance services utilization among childbearing women in Guinea

Variables	Model I (Antenatal care) AOR (CI)	Model II (Skilled birth attendance) AOR (CI)
**Age**		
15–24	1.29^*^ (1.03–1.62)	0.89 (0.69–1.13)
25–34	1.04(0.89–1.23)	1.00(0.84–1.18)
35+	Ref	Ref
**Mother’s level of education**		
No education	Ref	Ref
Primary	1.24^*^(1.01–1.52)	1.32^*^(1.04–1.68)
Secondary/Higher	1.70^***^(1.33–2.19)	1.83^***^(1.25–2.68)
**Partner’s level of education**		
No education	Ref	Ref
Primary	1.32^*^(1.04–1.68)	1.16(0.89–1.52)
Secondary/Higher	1.46^***^(1.22–1.75)	1.40^***^(1.11–1.76)
**Wealth**		
Poorest	Ref	Ref
Poorer	1.51^***^(1.23–1.84)	1.46^***^(1.22–1.74)
Middle	1.88^***^(1.53–2.29)	2.32^***^(1.93–2.79)
Richer	3.45^***^(2.72–4.38)	4.80^***^(3.72–6.19)
Richest	5.09^***^(3.70-7.00)	10.79^***^(6.64–17.51)
**Pregnancy intention**		
Unintended	Ref	Ref
Planned	1.50^***^(1.23–1.81)	1.25^*^(1.03–1.52)
**Religion**		
Christianity	Ref	4.29^***^(3.23–5.70)
Islam	1.65^***^(1.38–2.12)	Ref
Others	1.18(0.57–2.44)	4.09^***^(1.99–8.39)
**Marital status**		
Married	Ref	Ref
Cohabiting	1.46(0.89–2.40)	1.76(0.94–3.31)
**Place of residence**		
Urban	0.94(0.76–1.16)	3.00^***^(2.29–3.94)
Rural	Ref	Ref
**Parity**		
One birth	0.88(0.69–1.12)	1.58^***^(1.20–2.06)
Two births	0.86(0.70–1.05)	1.02(0.82–1.28)
Three births	0.89(0.74–1.07)	1.05(0.86–1.28)
Four or more births	Ref	Ref
**Employment status**		
Not working	-	1.07(0.91–1.25)
Working	-	Ref
**Health care decision making**		
Alone	1.53^***^(1.24–1.89)	1.87^***^(1.46–2.39)
Not alone	Ref	Ref
**Frequency of reading newspaper/magazine**		
Not at all	Ref	Ref
Less than once a week	0.78(0.52–1.16)	0.69(0.34–1.38)
At least once a week	1.97(0.61–1.54)	1.95(0.75–5.08)
**Frequency of listening to radio**		
Not at all	Ref	Ref
Less than once a week	1.30^**^(1.10–1.53)	1.05(0.87–1.26)
At least once a week	1.11(0.94–1.30)	1.19^*^(1.01–1.40)
**Frequency of watching television**		
Not at all	Ref	Ref
Less than once a week	0.94(0.78–1.14)	1.50^***^(1.21–1.85)
At least once a week	1.13(0.92–1.39)	1.69^***^(1.30–2.19)
**Sex of household head**		
Male	Ref	Ref
Female	1.20(0.97–1.48)	1.52^***^(1.20–1.92)
N	4917	4917
Pseudo R^2^	0.100	0.254

*aOR *adjusted Odds Ratio, 95% confidence intervals in brackets, ^*^p < 0.05, ^**^p < 0.01, ^***^p < 0.001; *Ref *reference category

Source: 2018 Guinea Demographic and Health Survey

With SBA, women with secondary/higher level of education (AOR = 1.83, CI = 1.25–2.68), those whose partners had secondary/higher level of education (AOR = 1.40, CI = 1.11–1.76), those in the richest wealth quintile (AOR = 10.79, CI = 6.64–17.51), those with planned pregnancies (AOR = 1.25, CI = 1.03–1.52), and Christian women (AOR = 4.13, CI = 3.17–5.39) were more likely to utilize the services, compared to women with no formal education, those whose partners had no formal education, those from poorest wealth quintile, those who had unintended pregnancies, and Muslims women respectively. Again, women in urban areas (AOR = 3.00, CI = 2.29–3.94), women with one birth (AOR = 1.58, CI = 1.20–2.06), those who take healthcare decisions alone (AOR = 1.87, CI = 1.46–2.39), those who read newspaper at least once a week (AOR = 1.19, CI = 1.01–1.40), those who watched television at least once week (AOR = 1.69, CI = 1.30–2.19), and those in female-headed households (AOR = 1.52, CI = 1.20–1.92) had higher odds of SBA service utilization, compared to women in rural areas, those with four or more births, those who do not take healthcare decisions alone, those who neither read newspaper nor watched television at all, and those in male-headed households respectively.

## Discussion

This study sought to assess ANC and SBA and its associated factors among childbearing women in Guinea. The study results revealed a direct association between increasing levels of education and utilisation of both ANC and SBA services. Specifically, women who had at least secondary level of education and those whose partners had at least secondary level of education showed higher probability of using both ANC and SBA services, relative to their counterparts with lower levels of education. Previous studies in developing countries such as Ethiopia [18, 10] and Lao [19]also found a strong association between couple’s level of education and ANC utilisation. Similar studies in Ethiopia [20–22], Guinea-Bissau [23], Nigeria [24], and northern parts of Ghana [25] revealed educational attainment as a predictor of utilization of SBA services. Generally, attainment of higher education has been noted as a strong predictor of maternal healthcare utilization [16, 26, 27]. Mezmur et al. [22] and Gebresilassie et al. [28] noted that higher education exposes women to knowledge on health issues which may positively influence their uptake of maternal healthcare services. Empirical evidence shows that educated women are more receptive to new health-promoting ideas which reinforce the demand side of health [29]. Women’s education develops literacy, which in turn is connected to an array of positive health outcomes because of “health literacy”, theorized as “the degree to which individuals have the capacity to obtain, process and understand basic health information and services needed to make appropriate health decisions” [30]. This educational attainment heightens women’s aptitude to identify sickness symptoms, use prevention services and better self-management of disease and the need to seek appropriate health care [31]. Significantly, women’s education bridges the somewhat traditional power distance between them and their male counterparts, especially in patriarchal sub-Saharan African societies by facilitating their empowerment, and thus, considerable decision-making power at the household and community level [32]. According to some researchers [33–35], women are capable or have greater autonomy to make constructive decisions about their own and that of their children’s health when well educated. For example, less empowered women in north-eastern Ghana were unable to choose a delivery place, decide on childcare health issues and even their own dietary needs [36]. Higher education makes women more confident and gives them the ability to take wise health decisions [20]. The other finding that women’s partners’ educational attainment strongly predicts maternal healthcare utilization highlights the significant roles men play in their partners’ health decision-making [25]. Thus, partner’s education may play similar supporting roles toward women’s access to ANC and SBA services. For example, some studies have emphasized that male involvement in women’s health needs promotes their maternal and reproductive healthcare choices and utilization [37–41]. Prakash, Swain, and Negi [42] noted that when husbands recognize the significance of women’s healthcare needs, related decisions are urgently addressed.

The study also identified wealth status as a predictor of utilization of both ANC and SBA services. Women within the richest wealth quintile specifically had higher propensity of utilizing maternal healthcare services, compared to their counterparts from the poorest wealth quintile, a finding that is consistent with previous studies in other countries [16, 17]. Some studies have specifically found higher likelihood of utilization of ANC services among women in the richest wealth quantile [43, 18, 10, 44], with similar observations on SBA utilization [21, 22, 27, 45]. In Guinea, the main reason for the low likelihood of maternal healthcare utilization among women from poorer households could not be the cost associated with healthcare. This is because the Ministry of Health of Guinea has since 2010 provided free obstetric care services, including antenatal care and health facility delivery [46]. However, despite the availability of free maternal healthcare services in Guinea, women from poor homes are likely to face other barriers, such as cost of transportation, which may obstruct their uptake of ANC and SBA services. For poor households, priority may probably be given to basic daily needs instead of healthcare expenses because of lack of adequate financial resources [47, 48]. Wealthier women, on the other hand, will certainly not face these barriers, as they have money to pay for the maternal healthcare services, even if they were costly, and can also take care of other financial obligations which could prevent poorer women from accessing maternal healthcare services. Appropriate service delivery methods that include local community-based interventions that target vulnerable poor women groups are required [49].

Religion and intention of pregnancy as strong predictors of maternal healthcare services utilization among women in Guinea were also noted. Specifically, the study showed a significant association between being a Muslim woman and a Christian woman and uptake of ANC and SBA services respectively. This finding corroborates other findings in some previous studies in northern Ghana [25, 50] and rural Nigeria [24] which revealed an association between Christianity and SBA uptake. Conversely, this finding contradicts other findings [22] that did not find religion as a predictor of SBA uptake. Although no clear explanation can be attributed to this pattern in the current findings, we speculate that quite a number Muslim women are betrothed to very influential Muslim men at younger adult age so are more compelled to seek ANC because they lack personal experience related to maternal practices. However, when these Muslim women are due for delivery, strong religio-cultural norms allow these women to deliver at homes through the help of untrained traditional birth attendants through spousal and extended family endorsement. Such indigenous maternal practices are minimal with Christian women, though may exist. Despite this finding, there is the need for future qualitative research to understand more about the role of religion in maternal healthcare utilization. There is the need to formulate and disseminate particular culturally appropriate messages targeting women and families based on their religions and offer home-based care to those who have challenges using ANC and SBA. These interventions can be complemented with community-based interventions that focus more on awareness creation that could help modify some cultural practices that might have outlived their usefulness, especially among less educated and those with low socioeconomic status[49].

Similar to previous research [51, 52], women with intended pregnancies were more likely to patronize ANC and SBA services, compared with their counterparts with unintended pregnancies. This finding confirms the findings of Dutamo et al. [51] and Tesfaye et al. [52] while the link between unintended pregnancy and low uptake of maternal healthcare services seems unclear, some researchers have speculated some reasons for this association. Parkhurst et al. [53], for instance, indicated that this association could be suggestive of women’s inability to control issues related to both their reproductive health and household resources. Tesfaye et al. [52] also noted that women who unintentionally get pregnant may consider having abortion, which prevents their subsequent use of maternal healthcare services. Alternatively, women with unwanted pregnancies might be reluctant to seek ANC and SBA services.

Healthcare decision-making also strongly predicted uptake of maternal healthcare services. Specifically, women who take healthcare decisions alone were more probable to use ANC and SBA services compared to those who do not take healthcare decisions alone. This finding is consistent with the findings of Ousman et al. [18] who found women’s decision making capacity as a strong predictor of ANC uptake in Ethiopia. Similarly, Ameyaw et al. [54] noted that women’s autonomy in making healthcare decisions positively affect uptake of SBA services in Ghana. This finding suggests that women’s ability to make decisions concerning their health could positively affect their uptake of maternal healthcare services. Despite our finding that partners’ education plays a role in women’s healthcare decisions and thus in their utilization of ANC and SBA, the finding that women who take healthcare decisions alone were more probable to use ANC and SBA services compared to those who do not take healthcare decisions alone is an indication that the level of education of the partner may not always have an influence on a woman’s autonomy but rather her own education.

Besides, women who listen to radio less than once a week have higher probability of using ANC services and that those who watch television less than once a week are more likely to use SBA services than those who do not watch television or read newspaper at all. Some previous studies [55, 56, 18, 57] suggest that radio, television, and newspaper could be channels through which health information reach women. Therefore, women who use such media easily get access to information on the importance of maternal healthcare services, which could influence them to use maternal healthcare services such as ANC and SBA [58]. Zamawe et al. [59] revealed how mass media campaign positively affected the use of health services including ANC, in Malawi. Hence, women exposed to media are more likely to have more information, knowledge, positive attitudes and may value the importance of ANC and SBA services by utilizing them. Residential variation in the utilization of SBA services in Guinea was also established in the current study. Context-specific factors such as lack of competent personnel, quality services as well as varied socio-cultural norms and poor socio-economic status of women may restrict SBA utilization in the rural setting compared to their urban counterparts. For example, limited availability of transport, traveling longer distances to access health care facilities, and the cost of maternal services may serve as barriers for rural women [60, 61]. A comprehensive community-level interventions that consider residential homogeneity regarding infrastructure (e.g., health facilities, roads) and socioeconomic empowerment (e.g., educational and vocational training) could boost women health-promoting behaviours and their access to maternity healthcare services.

### Strengths and limitations

The present study comes with some strengths and limitations. First, the use of a nationally representative data allows for generalizability of the findings to all childbearing women in Guinea. Second, using higher order statistical models such as the binary logistic regression ensured a rigorous analysis of the data, thus provides credibility to the findings obtained. For limitations, the study used a cross-sectional research design that limits causal relations between the studied variables. This study also suffers from recall biases which often characterize DHS data. In the present study, the recall bias may result from the retrospective nature of self-reporting the use of ANC and SBA services.

## Conclusions

Women with at least secondary level of education, wealthier, Muslim or Christian, those with intended pregnancies, health decision-making capacity and had media exposure were associated with uptake of ANC and SBA services in Guinea. These findings suggest the need to design community-based interventions (e.g., miniature local ANC clinics, early screening services) that prioritize women’s education and vocational training, media accessibility, especially among the poor, and those residing in rural settings. Such interventions should not ignore the influence of other socio-cultural norms that hinder the utilization of ANC and SBA services in Guinea.

## Data Availability

Data is available on https://dhsprogram.com/what-we-do/survey/survey-display-437.cfm.

## Abbreviations

ANC: Antenatal Care
AOR: Adjusted Odds Ratio
CI: Confidence Interval
GDHS: Guinea Demographic and Health Surveys
WHO: World Health Organization
SDG: Sustainable Development Goal
SSA: Sub-Saharan Africa
